# Thyroid-Like Follicular Carcinoma of the Kidney in a Young Patient with History of Pediatric Acute Lymphoblastic Leukemia

**DOI:** 10.1155/2014/313974

**Published:** 2014-07-15

**Authors:** William W. Wu, Julia T. Chu, Ali Nael, Sherif A. Rezk, Stephen G. Romansky, Lisa Shane

**Affiliations:** ^1^Department of Pathology & Laboratory Medicine, University of California Irvine Medical Center, Orange, CA 92868, USA; ^2^Department of Pathology, Long Beach Memorial Medical Center, 2801 Atlantic Avenue, Long Beach, CA 90801, USA

## Abstract

Thyroid-like follicular carcinoma of the kidney (TLFCK) is a rare histological variant of renal cell carcinoma not currently included in the World Health Organization classification of renal tumors. Only 24 previous cases of TLFCK have been reported to date. We report a case of TLFCK in a 19-year-old woman with history of pediatric acute lymphoblastic leukemia. This patient is the youngest with TLFCK to be reported to date and the first with history of lymphoblastic leukemia. The development of TLFCK in a young patient with history of lymphoblastic leukemia is interesting and suggests that genes involved in leukemogenesis may also be important for TLFCK pathogenesis. Recognition of TLFCK is important to distinguish it from other conditions that show thyroid-like features, as a misdiagnosis can result in adverse patient care.

## 1. Introduction

Thyroid-like follicular carcinoma of the kidney (TLFCK), also known as thyroid follicular carcinoma-like tumor of the kidney or thyroid follicular carcinoma-like renal tumor, is a rare and recently described subtype of renal cell carcinoma (RCC) not included in the 2004 World Health Organization (WHO) classification of renal tumors [1]. First described in 2004 [2], only 24 cases have been described in the literature to date (Table 1) [3–21].

We report a case of TLFCK arising in a 19-year-old woman with history of childhood T-cell acute lymphoblastic leukemia (T-ALL). This patient is the youngest with TLFCK to date and the first with history of lymphoblastic leukemia. Atypical features such as the presence of isolated tumor cells and renal capsular invasion with cyst formation were also identified. Isolated tumor cells in TLFCK have not been previously described, to the best of our knowledge. A recent study showed that the mixed-lineage leukemia/trithorax homolog (MLL) gene is overexpressed in TLFCK when compared to clear cell and chromophobe RCC [6]. MLL is a recurring translocation found in a number of hematologic malignancies including lymphoblastic lymphomas and leukemias. The finding of TLFCK in a young patient with a history of lymphoblastic leukemia is intriguing and suggests a potential role of genes implicated in leukemogenesis in the development of TLFCK.

## 2. Case Report

A 19-year-old woman presented with right sided abdominal pain. Her past medical history was significant for T-ALL, diagnosed at 5 years of age. Flow cytometry and immunophenotyping on bone marrow showed the leukemic cells to be CD2−, CD7+, terminal deoxynucleotidyl transferase (Tdt)+, CD10+, and CD34+, consistent with T lymphoblasts. Her leukemia has remained in remission since treatment.

**Figure 1:**
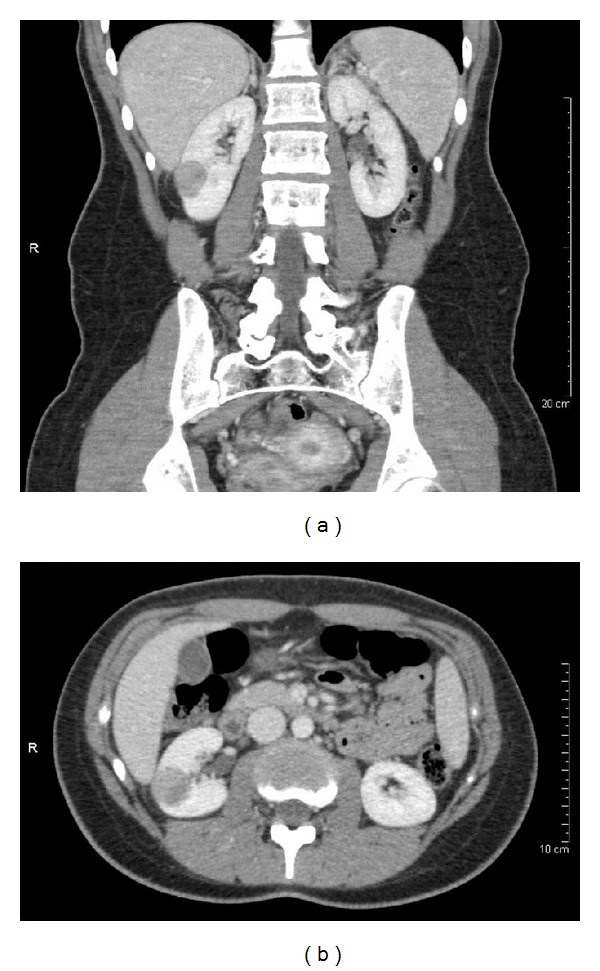
Imaging of TLFCK. (a) Coronal and (b) axial computed tomography with contrast showing a heterogeneous enhancing right renal mass.

Abdominal computed tomography (CT) scan showed a 2.8 × 2.3 × 2.3 cm heterogeneous, hyperdense, and partially exophytic lesion in the right lower kidney (Figure 1). No evidence of renal vein involvement, lymphadenopathy, or metastatic disease was identified. A skull-to-thigh positron emission tomography/computed tomography (PET/CT) scan demonstrated focal radiolabeled [18F]-2-fluoro-2-deoxy-D-glucose (18F-FDG) uptake in the right lower kidney tumor. No additional scintigraphic findings worrisome for malignancy were identified elsewhere in the body.

The patient subsequently underwent a needle core biopsy of the right renal mass followed by a right partial nephrectomy without complication. At 21 months after partial nephrectomy, there was no evidence of tumor recurrence or metastatic disease.

## 3. Materials and Methods

The biopsy and partial nephrectomy specimens were routinely fixed in 10% buffered formalin, embedded in paraffin, and serially sectioned. Routine staining with hematoxylin and eosin was performed. Immunohistochemical staining was performed using the avidin-biotin-peroxidase complex method on a BenchMark ULTRA processor (Ventana Medical Systems, Tucson, AZ, USA). Primary antibodies used include those for pancytokeratin (AE1/AE3), cytokeratin 7 (CK7), epithelial membrane antigen (EMA), thyroid transcription factor-1 (TTF-1), thyroglobulin (TG), paired box gene 2 (PAX-2), PAX-8, CD10, CD56, S-100 protein, carcinoembryonic antigen (CEA), smooth muscle myosin heavy chain (SMMHC), Wilms tumor-1 (WT-1), vimentin, and Ki-67. Appropriate positive and negative controls were used for each antibody.

## 4. Results

### 4.1. Renal Biopsy

**Figure 2:**
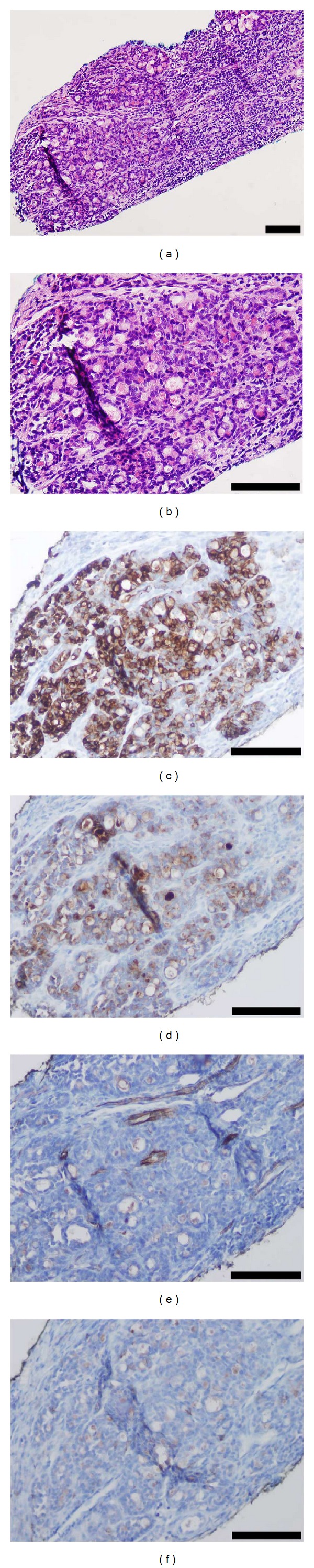
Histologic and immunophenotypic features of TLFCK on biopsy. (a) Low power view of biopsy showed an atypical area with microfollicles surrounded by chronic inflammatory reaction. (b) High power view demonstrated microfollicles with eosinophilic material filling tubular lumens. ((c) and (d)) Immunohistochemical staining of tumor cells showed reactivity for (c) CK7 and (d) EMA. ((e) and (f)) Tumor cells lacked immunoreactivity for (e) WT-1 and (f) CD56. Scale bars represent 100 *μ*m.

Needle core biopsy of the right renal mass demonstrated a focal atypical tubular epithelial cell proliferation (Figure 2(a)). The tubular epithelial cells in this focus possessed enlarged and hyperchromatic nuclei and enclosed small tubular lumens filled with dense eosinophilic material (Figure 2(b)). Immunohistochemical staining showed that the atypical epithelial cells were immunoreactive for CK7 (Figure 2(c)) and EMA (Figure 2(d)) while negative for WT-1 (Figure 2(e)) and CD56 (Figure 2(f)).

### 4.2. Partial Nephrectomy Specimen

**Figure 3:**
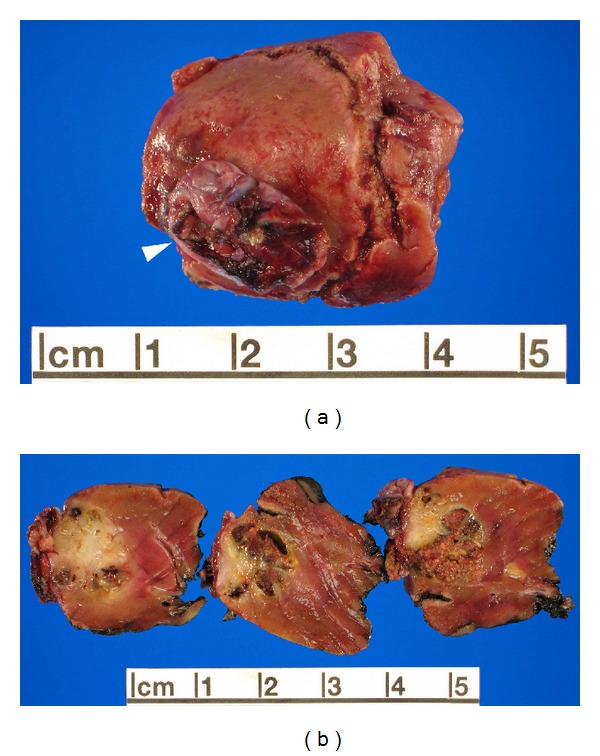
Gross appearance of TLFCK. (a) Partial nephrectomy specimen with ruptured cyst (arrowhead) at the renal capsular surface. (b) Sectioning of specimen reveals a tan-brown partially cystic mass with focal hemorrhage, extending to the ruptured cyst at the capsular surface.

The partial nephrectomy specimen measured 3.2 × 2.7 × 2.7 cm (Figure 3(a)). A ruptured cyst measuring 1.7 cm in greatest dimension was identified on the renal capsular surface (arrowhead, Figure 3(a)). Serial sectioning of the specimen revealed an ill-defined, tan-brown, and partially cystic tumor with focal hemorrhage measuring 2.0 × 1.5 × 1.3 cm (Figure 3(b)). The tumor focally penetrated through the renal capsule at the site of the ruptured cyst. The surgical margins were free of tumor. The tumor was entirely processed.

**Figure 4:**
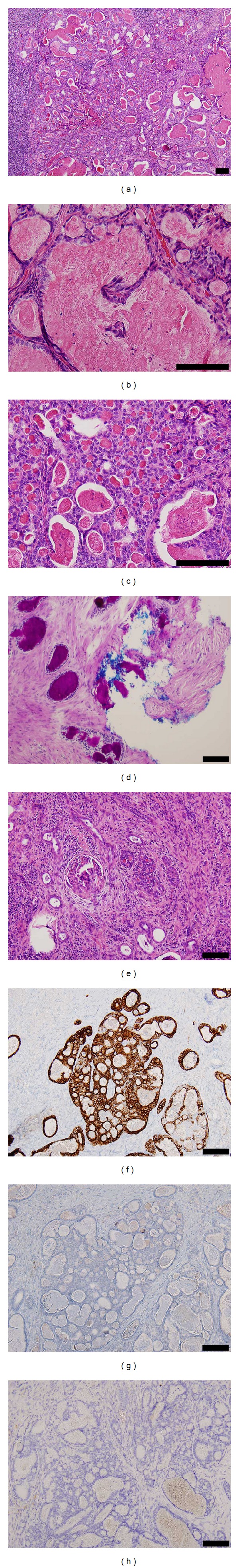
Histologic and immunophenotypic features of TLFCK. (a) Low power view of tumor demonstrated follicles of variably sized and shaped follicles filled with dense eosinophilic material. (b) Papillary-like structure extending into lumen of a macrofollicle. (c) High power view of microfollicular area. (d) Tumor extended to renal capsular surface (blue ink), with periodic-acid-Schiff (PAS) positive diastase resistant material identified within follicular lumens. (e) A focus of isolated tumor cells was located 1.8 mm from the main tumor. ((f)–(h)) Immunohistochemical staining of tumor cells showed reactivity for (f) CK7 and no reactivity for (g) TTF-1 or (h) thyroglobulin. Scale bars represent 100 *μ*m.

Microscopically, sections of the tumor revealed epithelial follicular structures varying in size and shape, surrounded by fibrotic stroma with a dense chronic inflammatory reaction (Figure 4(a)). The tumor compressed adjacent renal parenchyma where sclerotic glomeruli were occasionally seen. Focally, the neoplastic epithelial follicles invaded the surrounding fibrotic stroma. In some areas of the tumor, large irregular macrofollicular structures filled with eosinophilic proteinaceous material were present. Occasional pseudopapillary structures extending into macrofollicular lumens were identified (Figure 4(b)). In other areas, densely packed aggregates of microfollicles filled with eosinophilic proteinaceous material were observed (Figure 4(c)). The intraluminal proteinaceous material filling the follicles was periodic-acid-Schiff (PAS) positive and diastase resistant. The neoplastic epithelial cells surrounding the follicles have enlarged oval hyperchromatic nuclei at Fuhrman nuclear grade 2. Nuclear grooves, nuclear inclusions, optically clear nuclei, or mitoses were not seen. Focally, the neoplastic cells extended to the renal capsule (Figure 4(d)). In addition, an isolated focus of tumor cells measuring 0.2 mm in greatest dimension was located 1.8 mm from the main tumor (Figure 4(e)).

The neoplastic epithelial cells were diffusely and strongly immunoreactive for CK7 (Figure 4(f)), AE1/AE3, EMA, PAX-2, and PAX-8. The tumor cells were negative for TTF-1 (Figure 4(g)), thyroglobulin (Figure 4(h)), CD10, WT-1, SMMHC, CEA, and S-100. The Ki-67 proliferation index of the epithelial component was estimated at 5%. A diagnosis of TLFCK was rendered.

## 5. Discussion

We report a case of TLFCK in a 19-year-old woman with history of treated pediatric T-ALL. TLFCK is a rare and recently described histologic variant of RCC that is not included in the current WHO classification of renal tumors [1]. Twenty-five cases of TLFCK have been reported in the literature, including the current case (Table 1) [3–21]. Our patient is the youngest with TLFCK to date and the first with a history of childhood lymphoblastic leukemia.

Four cases of TLFCK were first described in abstract format by Amin et al. in 2004 [2] and later expanded in 2009 to include 6 cases [6]. An earlier report of a primary renal thyroid-like carcinoma that was positive for thyroglobulin may represent metastatic papillary thyroid carcinoma, although a primary thyroid lesion was not detected after 30 months of follow-up [22]. TLFCK are usually found in middle-aged patients (mean: 41.8 years; range: 19–83 years), with 14 women and 11 men affected. The primary tumor sizes range from 1.9 to 16.0 cm (mean: 5.3 cm) and are solitary in all cases to date. There appears to be no correlation between tumor size and development of metastatic disease. Clinically, nearly half of the patients with TLFCK are asymptomatic (12/25 cases, 48%). In symptomatic patients, hematuria (8/25, 32%) and abdominal/flank pain (8/25, 32%) were the most common presentations. One patient presented with hypertension [14, 15]. Follow-up data was available for 22 patients. The majority of TLFCK cases followed an indolent course with metastatic disease observed in 4 patients [4, 6, 8, 20]. One patient developed a lung metastasis 2 months after initial diagnosis; however the metastasis is questionable given its reactivity for TTF-1 [4]. A second patient presented with a renal hilar lymph node metastasis [6]. Two other patients were found to have lymph node and lung metastases at the time of presentation [8, 20]. Except for one patient who died of complications of acute myeloid leukemia following chemotherapy [18], all patients with TLFCK were alive with a mean follow-up time of 21.3 months (range: 1–84 months). One patient remained disease-free 17 months after primary renal tumor resection but was then lost to follow-up [6]. No follow-up information was available for 3 patients [5, 11, 13].

The tumor in our case demonstrated features similar to previously reported TLFCK. Follicles of various sizes and shapes filled with eosinophilic material were found throughout the tumor, giving the tumor an appearance reminiscent of thyroid carcinoma. Amin et al.  [6] commented that the tumor in the only patient with metastatic disease in their series showed prominent areas of complex growth with increased variability in follicular size and shape, similar to our case. The present tumor also demonstrated invasion of the renal capsule with cyst formation as well as a microscopic cluster of isolated tumor cells located away from the main tumor. All previously described TLFCK have been solitary tumors [3–21]. The presence of isolated tumor cells in the kidney has not been previously reported in TLFCK, and its clinical significance is unclear.

The immunohistochemical profile of the current tumor is comparable to previously reported TLFCK cases (AE1/AE3+, CK7+, PAX-2+, PAX-8+, vimentin+, EMA+, TTF-1−, TG−, CD56−, WT-1−, CD10−, and CEA−). A review of available TLFCK cases shows that most tumors are positive for AE1/AE3 (100%, 9/9 cases), CAM5.2 (100%, 3/3), PAX-8 (100%, 2/2), EMA (92.3%, 12/13), CK19 (85.7%, 6/7), CK7 (78.3%, 18/23), and vimentin (69.6%, 16/23). Variable expression in TLFCK can be seen for CK34*β*E12 (50%, 3/6 cases), NSE (50%, 2/4), PAX-2 (40.0%, 4/10), CD10 (33.3%, 7/21), HBME-1 (33.3%, 1/3), galectin-3 (33.3%, 1/3), CK20 (30.8%, 4/13), CD99 (25%, 1/4), CD15 (16.7%, 1/6), AMACR (9.1%, 1/11), RCC marker (8.3%, 1/12), CD56 (7.1%, 1/14), and WT-1 (6.7%, 1/15). TLFCK are negative for TTF-1 (0/24 cases), TG (0/24), CD57 (0/10), synaptophysin (0/8), CD117 (0/7), CEA (0/5), chromogranin (0/5), and Ksp-cadherin (0/5). The Ki-67 proliferation index of the current tumor was low at 5%, similar to other TLFCK cases [5, 13], although three cases of TLFCK demonstrated Ki-67 proliferation indices of 20% or greater [16, 17, 21]. The significance of the Ki-67 proliferation index in TLFCK remains to be determined.

Twenty-four percent of TLFCK patients (6/25) had history of a previous malignancy (Table 1). Previous malignancies included osteosarcoma of the rib [6], adenocarcinomas of the colon [6] and prostate [10, 18], Hodgkin lymphoma [10], acute myeloid leukemia [18], and T-ALL in the current case. The relatively high percentage of TLFCK patients with previous malignancies suggests that genetic factors may play an important role in the development of TLFCK. Alternatively, chemotherapeutic regimens used to treat previous malignancies may predispose to the development of TLFCK. Recently, a case of TLFCK was described in a patient with autosomal dominant polycystic kidney disease (ADPKD) [19]. ADPKD is associated with the development of other forms of RCC; however the association between ADPKD and TLFCK is still unclear. Similarly, the association between prior malignancies and/or chemotherapeutic treatment with the development of TLFCK remains to be determined.

Various genetic abnormalities have been identified in TLFCK [3, 4, 6]. Using comparative genomic hybridization analysis, Soo et al. [3] identified losses of chromosomes 1p36, 3 and 9q21–33 and gains of 7q36, 8q24, 12, 16, 17p11-q11, 17q24, 19q, 20q13, 21q22.3, and Xp. William et al. [4] showed losses of chromosomes 1, 3, 7, 9p21, 12, 17, and X by fluorescent in situ hybridization (FISH). These genetic abnormalities are distinct from the ones observed in clear cell or chromophobe RCC. In their series of TLFCK, Amin et al. [6] compared the gene expression profiles of TLFCK to clear cell and chromophobe RCC and found that TLFCK overexpressed 135 genes and underexpressed 46 genes. Interestingly, it was found that the MLL gene was overexpressed 2.5-fold in TLFCK when compared to clear cell and chromophobe RCC. MLL is a recurring translocation in hematologic malignancies including lymphoblastic lymphomas and leukemias and can be found in 8% of T-ALL cases [23]. FISH analysis on a formalin fixed paraffin embedded section of tumor from our patient failed to demonstrate translocations or amplifications of the MLL gene. However, the FISH assay could not eliminate the possibility of other structural abnormalities involving MLL or other genes and chromosomes. Genes involved in lymphoma and leukemia pathogenesis may play an important role in the development of TLFCK. Of note, two other TLFCK patients also had previous history of hematologic disorders [10, 18]. Further studies are necessary.

The differential diagnosis of TLFCK includes renal metastases of thyroid carcinoma, other primary renal tumors with thyroid-like features, and thyroidization of renal tubules. Of primary concern is thyroid carcinoma metastatic to the kidney. As the management of metastatic thyroid carcinoma and TLFCK differ greatly, misdiagnosis can result in suboptimal patient management.

Renal metastases from primary thyroid carcinoma are relatively uncommon with fewer than 40 cases reported to date [24–56]. Unlike TLFCK which usually presents as a solitary tumor, most patients with metastatic thyroid carcinoma have obvious thyroid tumors and widely disseminated disease at the time of presentation. Metastatic thyroid carcinoma should express TTF-1 and TG, both of which should be negative in TLFCK. Renal metastases from thyroid carcinoma arising in teratomas or ectopic thyroid tissue should also be considered for completeness. However, these possibilities are very remote and no cases have yet been reported.

Thyroid-like features can occur in other primary renal tumors, as well as in extra-renal neoplasms. Specifically, thyroid-like features have been documented in upper tract urothelial carcinoma [57], carcinoma of the breast [58, 59], intrahepatic cholangiocarcinoma  [60, 61], endolymphatic sac tumor   [62, 63], and plasmacytoma   [64]. In the kidney, thyroid-like features have been described in papillary RCC   [65–67]. Interestingly, in one case of metastatic papillary RCC, the metastatic tumor nodules in the patient's scalp also showed thyroid-like features [67]. Distinguishing TLFCK from papillary RCC with thyroid-like features may be difficult. Papillary RCC with thyroid-like features are composed of areas that resemble classic papillary carcinoma admixed with thyroid-like areas. In contrast, TLFCK are composed exclusively of thyroid-like macro- and microfollicles containing eosinophilic colloid-like material. Follicle-like spaces containing eosinophilic material can also be seen in metanephric adenomas and renal oncocytomas, although these features are typically minor components of these tumors [68, 69].

Similar to TLFCK, renal neuroendocrine (carcinoid) tumors are rare. Renal carcinoids have an increased incidence in horseshoe kidney [70]. Renal carcinoids with areas of pseudoglandular formation containing eosinophilic secretions can have a thyroid-like appearance. Immunohistochemical staining with neuroendocrine markers (chromogranin, synaptophysin, and CD56) is useful in differentiating renal carcinoids from TLFCK, the latter being negative for these markers. Interestingly, two TLFCK cases with Ki-67 proliferation indices of 20% or greater expressed neuron specific enolase (NSE) [16, 17].

Thyroidization of the renal tubules can occur in end stage renal disease, chronic pyelonephritis, or obstructive nephropathy. Unlike TLFCK which typically presents as a discreet mass, thyroidization of the renal tubules is often diffuse and involves bilateral kidneys.

In conclusion, TLFCK is a rare and recently described variant of renal carcinoma not included in the current WHO classification of renal tumors. Current consensus from the International Society of Urological Pathology (ISUP) is to not recommend TLFCK as a new WHO histological classification given the limited number of cases available for review [71]. Although the prognosis of most cases is favorable, TLFCK has uncertain malignant potential and metastatic disease can occur [4, 6, 8, 20]. We describe the presence of isolated tumor cells in TLFCK which has not been described previously. The pathogenesis of TLFCK is currently unknown and further studies are hindered by the limited number of these rare tumors. Although no translocations nor amplifications involving the MLL gene were identified in our case, the development of TLFCK in a young patient with history of T-ALL is notable and suggests that genetic factors predisposing to the development of lymphoma and leukemia may also be responsible for the pathogenesis of TLFCK.

## Figures and Tables

**Table 1:** Clinicopathological features of thyroid-like follicular carcinoma of the kidney.

Case	Reference	Age (years)/gender	Clinical presentation	Location	Size (cm)	Previous malignancy	Follow-up
1	Jung et al. [3]	32/F	Incidental	Right kidney, mid-pole + lower pole	11.8	nd	6 months, ANED
2	Sterlacci et al. [4]	29/F	Incidental	Left kidney, mid-pole	5.0	nd	60 months, ANED^a^
3	He et al. [5]	22/F	Painless hematuria	Left kidney	8.0	nd	nd
4	Amin et al. [6]	53/F	Incidental	Right kidney, mid-pole	2.1	Primary osteosarcoma of rib, s/p chemotherapy	54 months, ANED
5	Amin et al. [6]	29/F	Incidental	Right kidney, upper pole	1.9	nd	84 months, ANED
6	Amin et al. [6]	45/M	Incidental	Right kidney, lower pole	3.5	nd	17 months, ANED, then lost to follow-up^b^
7	Amin et al. [6]	83/M	Incidental	Left kidney, lower pole	2.1	Primary colonic adenocarcinoma, s/p chemotherapy	48 months, ANED
8	Amin et al. [6]	35/M	Incidental	Right kidney, mid-pole	3.0	nd	20 months, ANED
9	Amin et al. [6]	50/M	Incidental	Right kidney, mid-pole	4.0	nd	7 months, ANED
10	Xu and Zang [7]	36/F	Hematuria	Left kidney, mid-pole + lower pole	10.0	nd	12 months, ANED
11	Dhillon et al. [8]	34/F	Flank pain, gross hematuria	Right kidney, mid-pole	6.2	nd	3 months^c^
12	Khoja et al. [9]	31/F	Flank pain, gross hematuria, weight loss	Left kidney, upper-mid-pole	4.0	nd	21 months, ANED
13	Alessandrini et al. [10]	76/M	Gross hematuria	Left kidney, upper pole	4.5	Prostatic adenocarcinoma at age 71 y, s/p radical prostatectomy and adjuvant radiotherapy	11 months, ANED
14	Alessandrini et al. [10]	41/F	Incidental	Right kidney, lower pole	4.3	Hodgkin lymphoma, s/p splenectomy and chemoradiotherapy	4 months, ANED
15	Dhillon et al. [11]	34/M	Flank pain	Left kidney	2.8	nd	nd
16	Malde et al. [12]	29/F	Abdominal pain	Left kidney, lower pole	6.5	nd	4 months, ANED
17	Wu et al. [13]	26/F	Incidental	Right kidney	4.0	nd	nd
18	Wu et al. [14],Wu [15]	25/F	Hypertension	Right kidney, upper pole	2.5	nd	18 months, ANED
19	Li et al. [16], Lin et al. [21]	65/M	Hematuria, flank pain	Right kidney, mid-lower pole	8.0	nd	15 months, ANED
20	Tang et al. [17]	66/M	Gross hematuria, flank pain	Right kidney	16.0	nd	20 months, ANED
21	Berens et al. [18]	58/M	Incidental autopsy finding	Left kidney	3.0	Acute myeloid leukemia + prostatic adenocarcinoma	Died^d^
22	Volavšek et al. [19]	34/M	Abdominal pain	Left kidney, lower pole	5.5	nd	6 months, ANED
23	Vicens et al. [20]	34/F	Gross hematuria, flank pain	Right kidney, interpolar	6.2	nd	ASD^e^
24	Lin et al. [21]	59/M	Incidental	Right kidney, mid-lower pole	6.0	nd	1 month, ANED
25	Current case	19/F	Abdominal pain	Right kidney, lower pole	2.0	Acute T-lymphoblastic leukemia at age 5 y, s/p chemotherapy	21 months, ANED

^a^Developed lung metastasis 2 months after initial diagnosis.

^b^Renal hilar lymph node metastasis at initial diagnosis.

^c^Lung and retroperitoneal lymph node metastases at initial diagnosis.

^d^Died of complications of acute myeloid leukemia after chemotherapy 18 days after hospitalization.

^e^Lung and lymph node metastases at initial diagnosis, on sunitinib malate with stable lung metastases and no evidence of local recurrence or new metastases.

F: female.

M: male.

ANED: alive with no evidence of disease.

ASD: alive with stable disease.

nd: not described.
